# Health at a crossroads: future of the European Union hangs in the balance

**DOI:** 10.1016/j.lanepe.2024.101018

**Published:** 2024-08-01

**Authors:** The Lancet Regional Health – Europe

In June, 185 million Europeans cast their votes to elect 720 Members of the European Parliament (MEPs), the world's only directly elected transnational assembly. Despite the significance of this election, the voter turnout was disappointing at 51%, ranging from less than 30% in Croatia and Lithuania to more than 80% in Belgium and Luxembourg. The centre-right European People's Party and the centre-left Socialists and Democrats remained the most voted, securing around 25% and 20% of seats, respectively. Meanwhile, far-right parties made considerable gains, particularly in large countries such as Germany and France, while the Greens/European Free Alliance and the liberals Renew Europe experienced substantial losses.

This shift towards a more right-leaning European Parliament raises crucial questions about the potential implications for public health in the European Union (EU). Will important health agendas lose momentum? There is a serious concern that health could be at risk of diminishing in priority on the EU political agenda. The newly right-leaning European Parliament, with parties inclined to view health as a national rather than a collective responsibility, might advocate for minimal EU-level involvement in health policies.

On June 21, 2024, the European Council approved the Draft Council Conclusions on the future of the European Health Union, urging the European Commission to prioritise health in its upcoming term. Paradoxically, just 6 days later, the same European Council adopted the EU strategic agenda for 2024–29, outlining the EU's priorities for the next 5 years, which significantly fails to prioritise health. Health is mentioned only three times in the document: in the context of strengthening resilience and preparedness for health emergencies, capacity building for technological development and pharmaceuticals, and cooperation to enhance access to medicines. This agenda overlooks crucial issues such as rising health inequity, the surge in non-communicable diseases—the leading cause of death in the EU—exacerbated by a rapidly aging population, increasing prevalence of mental health conditions, and the alarming shortage of health-care workers. However, the emphasis on climate action and transition to climate neutrality is encouraging, especially with fewer MEPs prioritising this area in the new mandate.

The health agendas over the next 5 years will depend on the newly elected Commissioners, notably the Commissioner overseeing health, led by Ursula von der Leyen, the second-term President of the European Commission. The evolving European political landscape might influence the selections, potentially favouring national interests over the collective wellbeing of Europe as a whole. Much will also depend on which EU policies are classified under the purview of health and who will lead their implementation. Expectations are high for continuity and enhancement of important health initiatives such as Europe's Beating Cancer Plan, the Pharmaceutical Strategy for Europe, and the EU Global Health Strategy, introduced in the previous mandate, although outcomes remain uncertain.

Commissioners and MEPs must prioritise health alongside all other agendas, recognising health-care expenditure as an investment rather than a cost. It is imperative for them to commit to developing a comprehensive strategy to prevent and tackle non-communicable diseases, foster the creation of healthy environments and sustainable food systems, and address the commercial and socioeconomic determinants of health. Additionally, they must champion the advancement of digital health literacy, digital health tools, and the ethical use of health data and artificial intelligence in health care. The EU should ensure that all policies consider their impacts on mental health, physical health, and wellbeing. Establishing a true European Health Union is essential for safeguarding the health of all Europeans and should be a fundamental long-term goal of this legislative term.

This is a defining moment for public health in the EU. The decisions made by this newly elected Parliament will either propel us towards a healthier, more united Europe or set us back, jeopardising the wellbeing of millions. The stakes could not be higher. Health is at a crossroads, and every eligible EU citizen must take responsibility for the future of their continent by actively participating in elections. A 51% voter turnout is discouraging and highlights the need for greater civic engagement. In democratic societies, voting is both a right and a responsibility. Exercising this right with a sense of pride and proactive commitment, not complacency, ensures that our voices are heard and shapes a healthier, more equitable future for all.

